# Design and Synthesis of Malonamide Derivatives as Antibiotics against Methicillin-Resistant *Staphylococcus aureus*

**DOI:** 10.3390/molecules23010027

**Published:** 2017-12-22

**Authors:** Jung-Chen Su, Yu-Ting Huang, Chang-Shi Chen, Hao-Chieh Chiu, Chung-Wai Shiau

**Affiliations:** 1 Institute of Biopharmaceutical Sciences, National Yang-Ming University, Taipei 11221, Taiwan; jjjaannee@hotmail.com (J.-C.S.); twilight-0824@hotmail.com (Y.-T.H.); 2 Faculty of Pharmacy, National Yang-Ming University, Taipei 11221, Taiwan; 3 Institute of Basic Medical Sciences, National Cheng Kung University, Tainan 70101, Taiwan; cschen@mail.ncku.edu.tw; 4 Department of Clinical Laboratory Sciences and Medical Biotechnology, National Taiwan University, Taipei 10617, Taiwan; 5 Department of Chemistry, Chung-Yuan Christian University, Chungli 32023, Taiwan

**Keywords:** malonamide, *Staphylococcus aureus*, antibiotics

## Abstract

Methicillin-resistant *Staphylococcus aureus* (MRSA) is a serious threat to humans. Most existing antimicrobial drugs, including the β-lactam and quinoxiline classes, are not effective against MRSA. In this study, we synthesized 24 derivatives of malonamide, a new class of antibacterial agents and potentiators of classic antimicrobials. A derivative that increases bacterial killing and biofilm eradication with low cell toxicity was created.

## 1. Introduction

Antibiotic resistance is a crucial issue in human health, and the launch of new antimicrobial drugs has become a rare event in the past few years [1,2,3]. The chronic misuse and overuse of β-lactam antibiotics has led to the development of drug-resistant pathogens. For example, *S. aureus* strains developed resistance to methicillin in the 1950s and soon spread to many hospitals worldwide [4]. *Staphylococcus aureus* is a virulent pathogen that causes a variety of infections, from minor infections of skin and soft tissue to life-threatening endocarditis, pneumonia and osteomyelitis [5,6]. Due to the limit of new antibiotics on the market, sources of antibacterial agents were explored from known drugs that were originally designed to treat symptoms other than bacterial infection. For example, statins, drugs for hypercholesterolemia, have been reported to reduce the virulence of *S. aureus* [7,8]. Phenothiazines, a class of antipsychotic agents, were discovered to exhibit antibacterial activity and potentiate antibiotics to eradicate extensively drug-resistant (XDR) *Mycobacterium tuberculosis* in patients [9,10]. Thus, known drugs represent an alternative source for the discovery and development of a novel series of antibacterial agents. 

**Figure 1:**
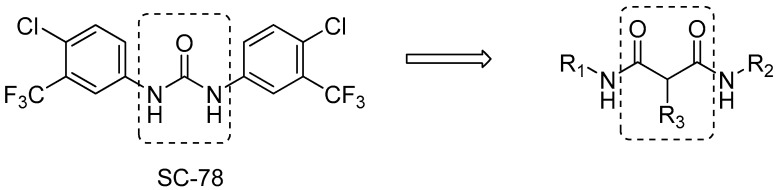
Chemical structure of SC-78 and scaffold of malonate derivatives.

Recently, we have performed screening assays against bacteria with our compound library which mostly contains kinase inhibitors and their derivatives. SC-78, a small molecule modified from regorafenib and sorafenib [11], multiple-kinase inhibitors, was shown to possess potent antimicrobial activities against *Staphylococcus aureus* and other *Staphylococcus* species [12] (Figure 1). From the pharmacological and medicinal chemistry perspective, the structure of SC-78 has crucial drawbacks. It consists of a stick central urea scaffold, on which the aniline groups are substituted with chloride and trifluoromethane. The symmetry of the whole structure makes SC-78 less soluble, which might influence its bioavailability. The aromatic rings give the molecule a planar structure, which is prone to stack the molecules through π–π interactions. Moreover, urea is a rigid scaffold, which reduces the interaction force between the whole molecule and its target. 

These drawbacks stimulated us to explore three modifications: replacement of the urea groups with isosteric amide linkers, replacement of the alkyl group with an alkenyl or benzyl group, and finally systematic modification of aniline derivatives with various functional groups on aromatic rings, thereby generating symmetric and asymmetric malonamide derivatives (Figure 1).

## 2. Results

**Scheme 1:**
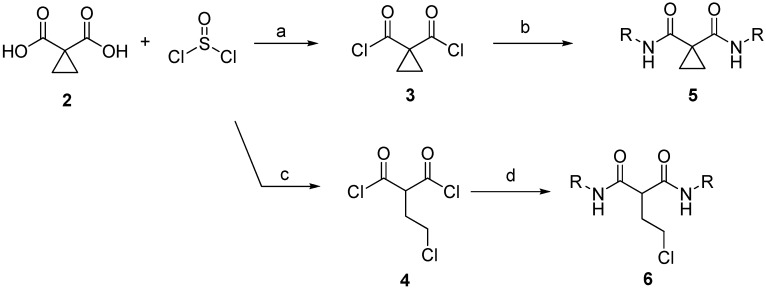
General synthesis of cyclopropanyl and ethyl chloride malonate derivatives. *Reagents and conditions*: (a) thionyl chloride; (b) phenyl amine, pyridine; (c) thionyl chloride, H_2_O; (d) phenyl amine, pyridine.

The dimethyl cyclopropane-1,1-dicarboxylate was considered a commercial starting skeleton replacing the urea structure. A hydrolysis step on dimethyl cyclopropane-1,1-dicarboxylate gave a quantitative amount of cyclopropane-1,1-dicarboxyilic acid **2**. For the convenience of the generation of the diacyl chloride from **2**, thionyl chloride was chosen as a reagent for the source of acyl chloride. At the same time, the ring-opening process of the cyclopropanyl group of **2** was found in the generation of acyl chloride with the present proton source. Finally, acylation of the resulting diacyl chlorides **3** and **4** with substituted anilines was designed to generate the final symmetrical products **5** and **6** (malonamide derivatives) (Scheme 1). 

**Scheme 2:**
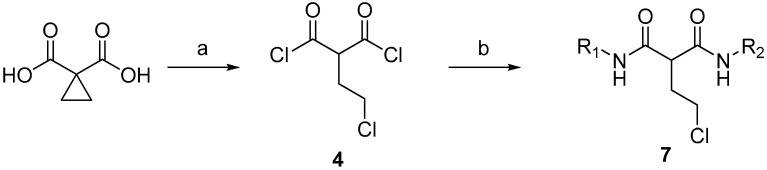
General synthesis of asymmetric malonate derivatives. *Reagents and conditions*: (a) thionyl chloride, H_2_O; (b) [i] R_1_NH_2_, pyridine; [ii] R_2_NH_2_, pyridine.

The strategy for the synthesis of the asymmetrical malonamide derivatives is shown in Scheme 2. Intermediate diacyl chloride was sequentially reacted with the first aniline substituent. After the half acylation reaction was completed, another aniline substituent was added to the mixture immediately for the completion of the asymmetrical malonate derivative **7**.

**Scheme 3:**
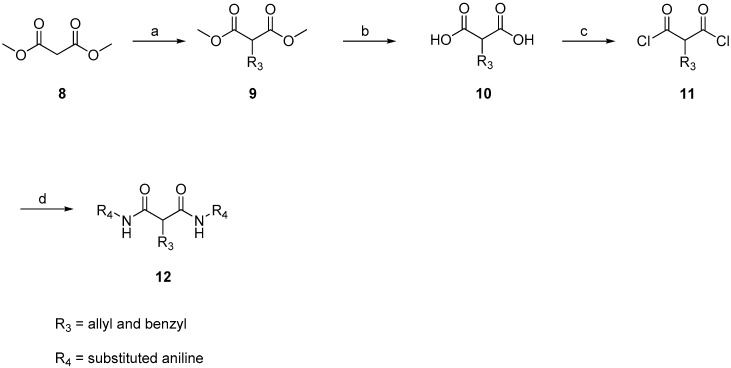
General synthesis of phenyl malonate derivatives. *Reagents and conditions*: (a) allyl bromide or benzyl bromide, K_2_CO_3_; (b) KOH, H_2_O; HCl; (c) thionyl chloride, H_2_O; (d) substituted aniline, pyridine.

Next, to explore the potency of ethane chloride in the malonamide derivative against *S. aureus*, ethane chloride was replaced by a cyclopropanyl, allyl or benzyl group, and the antibacterial activity and structure–activity relationship was analyzed. The strategy by which ethane chloride was replaced is shown in Scheme 3. Dimethyl malonate **8** was reacted with allyl chloride and benzyl chloride resulting in compound **9**. Hydroxylation of the methyl group by sodium methoxide led to compound **10**. Subsequently, the di-acid was converted to diacyl chloride **11** by thionyl chloride. Diacyl chloride was used to generate a series of amide-linked derivatives of malonamide **12**. 

Our efforts to design and synthesize novel anti-*Staphylococcus* molecules from SC-78 resulted in the development of a malonate scaffold as a new structural entity with suppressive activity against bacteria. These malonamide derivatives were assessed on *S. aureus* NCTC8325 by using the minimum inhibitory concentration (MIC) assay. The MIC values of all the malonamide derivatives are summarized in Table 1, Table 2 and Table 3. MIC values of each compound were determined by escalating doses, ranging from 0.125 to 64 mg/L.

A set of derivatives **13**–**26** were obtained in which chloride, nitro, hydroxyl, trifluoromethyl, ethynyl and trifluoromethoxyl substituents were attached to a phenyl ring on both sides of the malonate scaffold. The analysis of antibacterial activity of **17**, **20**, **21** and **22** with electron-donating substituents, such as hydroxyl and ethynyl on the phenyl ring, against *S. aureus* NCTC8325 demonstrated diminished suppressive activity (MIC > 8 mg/L). On the other hand, compounds **14**, **15**, **25** and **26** with electron-withdrawing substituents, such as trifluoromethoxyl and trifluoromethyl groups, increased the potency against *S. aureus* NCTC8325. The presence of the dichloride substituent in the phenyl ring led to an increase in activity relative to the monochloride substituent. These results suggest that electron-donating groups are less important than electron-withdrawing groups for antibacterial activity.

We next tested the hypothesis that the asymmetric substituents on both sides of the malonate scaffold might induce potent antibacterial activity with the symmetric substituents. Therefore, derivatives **28**, **29** and **30** were further synthesized and their antibacterial activity was evaluated. The results are shown in Table 2. The asymmetric malonamide derivatives exhibited almost equal antibacterial activity to the symmetric malonamide derivatives. 

Next, we tested whether ethane chloride is important in antibacterial activity. Cyclopropanyl, allyl and benzyl substituents were introduced into the center of the malonate scaffold to replace ethane chloride, resulting in a series of compounds as shown in Table 3. The loss of activity upon the replacement of ethyl chloride by the cyclopropanyl group may result from the bending of two carbonyl moieties, thereby losing interaction with the target. Derivatives with rigid benzyl and allyl groups show reduced activity, which is potentially connected to steric effects. We conclude that ethane chloride connected to malonate is crucial for the activity against *S. aureus* NCTC8325. 

To explore whether the synthesized compounds overcome antibiotic-resistant *Staphylococcus aureus*, we investigated the activity of compounds **26**, **27**, **28**, **29**, **33**, **36**, **37**, **38** and **39** in *S. aureus* NCTC8325. As shown in Table 4, the inhibitory potency of the compounds against MRSA ATCC33592 was almost the same as against *S. aureus* NCTC8325, except for compound **33**, which was neither effective against *S. aureus* NCTC8325 nor MRSA ATCC33592.

Compound **26** was selected for study of its pharmacological properties and evaluation of its drug-like potential. In-vitro study of antibacterial activity against various bacteria showed that **26** had excellent cell inhibitory effect on various antibiotic-resistant strains. Moreover, compound **26** inhibited cell growth on a K562 human erythroleukemic cell line with an IC_50_ of 20 mg/L. The selective ratio value of antibacterial versus cell line was over 40, indicating a good selectivity between humans and bacteria (Table 5).

Many clinical reports indicated that biofilm formation is an important factor contributing to *S. aureus* infections [13]. Bacteria in the biofilm usually exhibited less susceptibility to antibiotics, rendering treatment difficult [14]. To examine whether compound **26** was active against bacteria in biofilm, the minimal bacteria eradication concentrations (MBECs) of **26** and a wide range of antibiotics against MRSA ATCC33591 in biofilm were assessed. As the results show in Table 6, only compound **26** and rifampicin were active against MRSA 33591 in biofilm, but the MBEC values are much higher than their MIC values. Thus, we further tested whether **26** increased the susceptibility of MRSA toward these antibiotics. Among all the pairs of combinations, only rifampicin potentiated compound **26** in the MRSA ATCC33591 strain, while the others showed no synergistic effect (Table 6).

## 3. Discussion

There have been concerns about the threat of MRSA to global health in recent years because of the shortage of drugs that are effective against *S. aureus*, leading to lower survival in patients. *S. aureus* has also developed various mechanisms to evade the toxicity of antibiotic agents, such as β-lactamase and efflux pumps. The rate of development of antibiotic resistance is faster than the development of antibacterial agents with novel structures. After the latest antibacterial drugs, including linezolid, daptomycin and retapamulin, were approved for clinical application, no new antibiotic with a novel chemical entity has been introduced to the market. The gap in new antibiotic discovery highlights the need to develop new chemical backbones for new classes of antibiotic agents. In this study, we designed a series of agents from the lead compound, SC-78, with a urea backbone. Through pharmacochemical modification of the backbone, the malonate moiety exhibited a high potential and efficient chemical core for the development of a series of compounds. Our new compounds have several advantages: (1) At present, no resistance to compound **26** was observed to develop in vitro, suggesting a different inhibition mechanism from existing antibiotics and that it avoids the traditional antibiotic evasion strategies of MRSA; (2) in addition to providing a new core for an antibiotic agent, compound **26** alone exhibited high activity in vitro against several (at least three) MRSA strains. Compound **26** also showed excellent activity in killing MRSA inside biofilm. Interestingly, combinations of current antibiotics and **26** showed no synergy in eradicating MRSA in biofilm, with the exception of rifampicin; (3) we synthesized a set of malonamide derivatives via a two-step procedure. These efficient synthetic procedures applied commercially available chemicals and reagents to obtain a large set of malonamide derivatives that can be used in an animal study. From the structure–activity relationship analysis, a phenyl ring connected with an electron-withdrawing group, such as trifluoro and nitro, is important for biological activity. Replacement with a hydroxyl group resulted in loss of antimicrobiota activity. On the other hand, ethane chloride in the middle of the malonate moiety exhibited greater activity than allyl, benzyl and cyclopropanyl groups. 

In conclusion, we developed a short synthetic route for the preparation of a series of malonamide derivatives. Several agents showed promising antibacterial growth activity and repressed biofilm formation. Further exploration of the detailed mechanisms by which **26** overcome MRSA is ongoing. From the drug development point of view, the new scaffold described herein can guide the development of more potent agents and might provide therapeutic options for fighting infectious diseases.

## 4. Materials and Methods

### 4.1. Materials

Proton nuclear magnetic resonance (^1^H-NMR) spectra were recorded on Bruker Avance III (400 MHz, Bruker BioSpin, Rheinstetten, Germany) instruments. Chemical shifts are reported as δ values (ppm) downfield from internal deuterated chloroform, methanol and dimethyl sulfoxide of the indicated organic solution. Peak multiplicities are expressed as follows: s, singlet; d, doublet; t, triplet; q, quartet; dd, doublet of doublets; m, multiplet. Coupling constants (*J* values) are given in hertz (Hz). Reaction progress was determined by thin-layer chromatography (TLC) analysis on a silica gel 60 F254 plate (Merck KGaA, Darmstadt, Germany). Chromatographic purification was carried out on silica gel columns 60 (0.063–0.200 mm or 0.040–0.063 mm, Merck), basic silica gel. Commercial reagents and solvents were used without additional purification. Abbreviations are used as follows: CDCl_3_, deuterated chloroform; DMSO-*d*_6_, dimethyl sulfoxide-*d*_6_; EtOAc, ethyl acetate; MeOH, methanol; THF, tetrahydrofuran; EtOH, ethanol; DMSO, dimethyl sulfoxide. High-resolution mass spectra were recorded on a FINNIGAN MAT 95S mass spectrometer (Finnigan MAT, Bremen, Germany).

### 4.2. Chemical Synthesis 

#### 4.2.1. General Procedure for the Synthesis of **13**–**26**

Cyclopropane-1,1-dicarboxylic acid (1 equiv.) was slowly added to a mixture of thionyl chloride (14 equiv.) and one drop of water without any solution, and the reaction mixture was stirred at 80 °C for 16 h. The intermediate was cooled to ambient temperature and concentrated under reduced pressure. A mixture of aniline derivative (2.5 equiv.) and pyridine (1 equiv.) in anhydrous THF was added dropwise to an ice-cold solution of the intermediate in anhydrous THF (15–20 mL). The reaction mixture was stirred at ambient temperature for 2 h. The product was extracted with ethyl acetate three times and the combined organic extracts were washed with brine, dried over MgSO_4_, and concentrated. The crude mixture was purified by flash column chromatography (EtOAc/hexane = 1/4 to 1/1) to give **13** to **26** (yield: 4–30%).

*2-(2-Chloroethyl)-N^1^,N^3^-diphenylmalonamide* (**13**): ^1^H-NMR (400 MHz, MeOD-*d*_4_) δ 7.58 (d, *J* = 8.0 Hz, 4H), 7.32 (t, *J* = 8.0 Hz, 4H), 7.12 (t, *J* = 7.2 Hz, 2H), 3.75 (t, *J* = 7.2 Hz, 1H), 3.69 (t, *J* = 6.4 Hz, 2H), 2.48 (q, *J* = 6.4 Hz, 2H) ppm. HRMS calculated for C_17_H_17_ClN_2_O_2_ (M − H)^−^: 315.0895. Found: 315.0906.

*2-(2-Chloroethyl)-N^1^,N^3^-bis(3-(trifluoromethyl)phenyl)malonamide* (**14**): ^1^H-NMR (400 MHz, MeOD-*d*_4_) δ 8.05 (s, 2H), 7.80 (d, *J* = 8.0 Hz, 2H), 7.51 (t, *J* = 8.0 Hz, 2H), 7.40 (d, *J* = 8.0 Hz, 2H), 3.82 (t, *J* = 7.2 Hz, 1H), 3.70 (t, *J* = 6.8 Hz, 2H), 2.51 (q, *J* = 6.8 Hz, 2H) ppm. ^13^C-NMR (100 MHz, MeOD-*d*_4_) δ 169.4, 140.3, 132.1 (q, *J* = 32.0 Hz), 130.7, 125.4 (q, *J* = 269.9 Hz), 124.5, 121.8 (d, *J* = 3.6 Hz), 117.7 (d, *J* = 3.9 Hz), 54.0, 43.2, 34.2 ppm. HRMS calculated for C_19_H_15_ClF_6_N_2_O_2_ (M − H)^−^: 451.0643. Found: 451.0658.

*2-(2-Chloroethyl)-N^1^,N^3^-bis(4-(trifluoromethoxy)phenyl)malonamide* (**15**): ^1^H-NMR (400 MHz, MeOD-*d*_4_) δ 7.68 (d, *J* = 8.8 Hz, 4H), 7.22 (d, *J* = 8.8 Hz, 4H), 3.79 (t, *J* = 7.2 Hz, 1H), 3.68 (t, *J* = 6.4 Hz, 2H), 2.48 (q, *J* = 6.4 Hz, 2H) ppm. ^13^C-NMR (100 MHz, MeOD-*d*_4_) δ 169.2, 146.6, 138.4, 122.7, 122.6, 121.93 (q, *J* = 253.6 Hz), 53.9, 43.2, 34.3 ppm. HRMS calculated for C_19_H_15_ClF_6_N_2_O_4_ (M − H)^−^: 483.0541. Found: 483.0534.

*2-(2-Chloroethyl)-N^1^,N^3^-bis(3,5-dichlorophenyl)malonamide* (**16**): ^1^H-NMR (400 MHz, MeOD-*d*_4_) δ 7.65 (s, 4H), 7.17 (s, 2H), 3.76 (t, *J* = 7.2 Hz, 1H), 3.68 (t, *J* = 6.4 Hz, 2H), 2.46 (q, *J* = 6.4 Hz, 2H) ppm. ^13^C-NMR (100 MHz, DMSO-*d*_6_) δ 167.1, 140.9, 134.1, 122.9, 117.6, 52.4, 42.9, 31.6 ppm. HRMS calculated for C_17_H_13_Cl_5_N_2_O_2_ (M − H)^−^: 450.9336. Found: 450.9354.

*2-(2-Chloroethyl)-N^1^,N^3^-bis(3-ethynylphenyl)malonamide* (**17**): ^1^H-NMR (400 MHz, MeOD-*d*_4_) δ 7.75 (s, 2H), 7.58 (d, *J* = 7.6 Hz, 2H), 7.30 (t, *J* = 7.6 Hz, 2H), 7.21 (d, *J* = 7.6 Hz, 2H), 3.76 (t, *J* = 7.6 Hz, 1H), 3.68 (t, *J* = 6.8 Hz, 2H), 3.48 (s, 2H), 2.47 (q, *J* = 6.8 Hz, 2H) ppm. ^13^C-NMR (100 MHz, MeOD-*d*_4_) δ 169.2, 139.5, 130.0, 129.0, 124.6, 124.2, 121.8, 84.0, 78.8, 53.9, 43.2, 34.2 ppm. HRMS calculated for C_21_H_17_ClN_2_O_2_ (M − H)^−^: 363.0895. Found: 363.0892.

*2-(2-Chloroethyl)-N^1^,N^3^-bis(3-chlorophenyl)malonamide* (**18**): ^1^H-NMR (400 MHz, MeOD-*d*_4_) δ 7.77 (s, 2H), 7.45 (d, *J* = 8.4 Hz, 2H), 7.29 (t, *J* = 8.4 Hz, 2H), 7.11 (d, *J* = 8.4 Hz, 2H), 3.76 (t, *J* = 7.2 Hz, 1H), 3.68 (t, *J* = 6.8 Hz, 2H), 2.47 (q, *J* = 6.8 Hz, 2H) ppm. ^13^C-NMR (100 MHz, MeOD-*d*_4_) δ 169.2, 140.8, 135.4, 131.1, 125.4, 121.2, 119.4, 54.0, 43.2, 34.2 ppm. HRMS calculated for C_17_H_15_Cl_3_N_2_O_2_ (M − H)^−^: 383.0115. Found: 383.0114.

*2-(2-Chloroethyl)-N^1^,N^3^-bis(3-nitrophenyl)malonamide* (**19**): ^1^H-NMR (400 MHz, MeOD-*d*_4_) δ 8.63 (s, 2H), 8.02 (t, *J* = 8.0 Hz, 4H), 7.60 (t, *J* = 8.0 Hz, 2H), 3.82 (t, *J* = 7.6 Hz, 2H), 2.94 (t, *J* = 7.6 Hz, 2H) ppm. ^13^C-NMR (100 MHz, MeOD-*d*_4_) δ 166.9, 149.8, 140.3, 130.9, 127.7, 120.4, 116.6, 72.0, 42.4, 40.2 ppm.

*2-(2-chloroethyl)-N^1^,N^3^-bis(3-hydroxy-4-methylphenyl)malonamide* (**20**): ^1^H-NMR (400 MHz, MeOD-*d*_4_) δ 7.15 (d, *J* = 2.0 Hz, 2H), 7.01 (d, *J* = 8.0 Hz, 2H), 6.84 (dd, *J* = 8.0, 2.0 Hz, 2H), 3.74 (t, *J* = 7.6 Hz, 2H), 2.87 (t, *J* = 7.6 Hz, 2H), 2.14 (s, 6H) ppm.

*N^1^,N^3^-Bis(4-chloro-3-hydroxyphenyl)-2-(2-chloroethyl)malonamide* (**21**): ^1^H-NMR (400 MHz, MeOD-*d*_4_) δ 7.41 (s, 2H), 7.21 (d, *J* = 8.8 Hz, 2H), 6.93 (d, *J* = 8.8 Hz, 2H), 3.72–3.65 (m, 3H), 2.44 (q, *J* = 6.8 Hz, 2H) ppm.

*2-(2-Chloroethyl)-N^1^,N^3^-bis(3-hydroxyphenyl)malonamide* (**22**): ^1^H-NMR (400 MHz, MeOD-*d*_4_) δ 7.18 (s, 2H), 7.11 (t, *J* = 8.0 Hz, 2H), 6.96 (d, *J* = 8.0 Hz, 2H), 6.55 (d, *J* = 8.0 Hz, 2H), 3.72–3.65 (m, 3H), 2.45 (q, *J* = 6.8 Hz, 2H) ppm. 

*N^1^,N^3^-Bis(3,5-bis(trifluoromethyl)phenyl)-2-(2-chloroethyl)malonamide* (**23**): ^1^H-NMR (400 MHz, MeOD-*d*_4_) δ 8.30 (s, 4H), 7.72 (s, 2H), 3.82 (t, *J* = 7.6 Hz, 2H), 2.94 (t, *J* = 7.6 Hz, 2H) ppm. ^13^C-NMR (100 MHz, MeOD-*d*_4_) δ 167.1, 141.1, 133.3 (q, *J* = 33.2 Hz), 124.6 (q, *J* = 270.2 Hz), 121.7, 118.8 (q, *J* = 3.5 Hz), 71.8, 42.3, 40.1 ppm. HRMS calculated for C_21_H_13_ClN_2_O_2_F_12_ (M − H)^−^: 588.0474. Found: 588.0480.

*N^1^,N^3^-Bis(4-pentafluorosulfurphenyl)-2-(2-chloroethyl)malonamide* (**24**): ^1^H-NMR (400 MHz, DMSO-*d*_6_) δ 7.91–7.88 (m, 8H), 3.77 (t, *J* = 7.2 Hz, 2H), 2.88 (t, *J* = 7.2 Hz, 2H) ppm. ^13^C-NMR (100 MHz, DMSO-*d*_6_) δ 165.0, 148.1 (q, *J* = 16.1 Hz), 141.4, 126.6, 120.5, 71.1, 40.3 ppm. HRMS calculated for C_17_H_15_ClN_2_O_2_S_2_F_10_ (M − H)^−^: 568.0104. Found: 568.0109.

*2-(2-Chloroethyl)-N^1^,N^3^-bis(4-nitro-3-(trifluoromethyl)phenyl)malonamide* (**25**): ^1^H-NMR (400 MHz, MeOD-*d*_4_) δ 8.26 (s, 2H), 8.09–8.04 (m, 4H), 3.90 (t, *J* = 6.8 Hz, 1H), 3.73 (t, *J* = 6.4 Hz, 2H), 2.53 (q, *J* = 6.4 Hz, 2H) ppm.

*2-(2-Chloroethyl)-N^1^,N^3^-bis(4-chloro-3-(trifluoromethyl)phenyl)malonamide* (**26**): ^1^H-NMR (400 MHz, MeOD-*d*_4_) δ 8.12 (d, *J* = 2.8 Hz, 2H), 7.82 (dd, *J* = 8.8, 2.8 Hz, 2H), 7.54 (d, *J* = 8.8 Hz, 2H), 3.81 (t, *J* = 7.2 Hz, 1H), 3.70 (t, *J* = 6.8 Hz, 2H), 2.50 (q, *J* = 6.8 Hz, 2H) ppm. ^13^C-NMR (100 MHz, MeOD-*d*_4_) δ 169.3, 138.9, 133.3, 129.4 (q, *J* = 31.5 Hz), 127.5, 125.6, 124 (q, *J* = 271 Hz), 120.1 (q, *J* = 5.4 Hz), 54.1, 43.3, 34.1 ppm.

#### 4.2.2. General Procedure for Synthesis of Compounds **27**–**29**

Cyclopropane-1,1-dicarboxylic acid (1 equiv.) was slowly added to a mixture of thionyl chloride (14 equiv.) and one drop of water without any solution, and the reaction mixture was stirred at 80 °C for 16 h. The intermediate was cooled to ambient temperature and concentrated under reduced pressure. A mixture of aniline derivative (1.2 equiv.) and pyridine (1 equiv.) in anhydrous THF was added dropwise to an ice-cold solution of the intermediate in anhydrous THF (15–20 mL). After 30 min, another aniline derivative (1.8 equiv.) and pyridine (1 equiv.) was added to the mixture. The reaction mixture was stirred at ambient temperature for 2 h. The product was extracted with ethyl acetate three times and the combined organic extracts were washed with brine, dried over MgSO_4_, and concentrated. The crude mixture was purified by flash column chromatography (EtOAc/hexane = 1/4 to 1/1) to give **27**–**29** (yield: 20–45%).

*N^1^-(4-Chloro-3-(trifluoromethyl)phenyl)-2-(2-chloroethyl)-N^3^-(3,5-dichlorophenyl)malonamide* (**27**): ^1^H-NMR (400 MHz, MeOD-*d*_4_) δ 8.13 (s, 1H), 7.83 (d, *J* = 8.8 Hz, 1H), 7.65 (s, 2H), 7.55 (d, *J* = 8.8 Hz, 1H), 7.17 (s, 1H), 3.78 (t, *J* = 7.6 Hz, 1H), 3.69 (t, *J* = 6.4 Hz, 2H), 2.48 (q, *J* = 6.4 Hz, 2H) ppm. ^13^C-NMR (100 MHz, MeOD-*d*_4_) δ 169.3, 169.2, 141.7, 138.8, 136.1, 133.0, 129.3 (q, *J* = 31.3 Hz), 127.5, 125.5, 124.9, 124.1 (q, *J* = 270.4 Hz), 120.0 (q, *J* = 5.5 Hz), 119.3, 54.1, 43.2, 34.0 ppm. HRMS calculated for C_18_H_13_Cl_4_N_2_O_2_F_3_ (M − H)^−^: 485.9683. Found: 485.9679.

*N^1^-(3,5-Bis(trifluoromethyl)phenyl)-2-(2-chloroethyl)-N^3^-(3,5-dichlorophenyl)malonamide* (**28**): ^1^H-NMR (400 MHz, MeOD-*d*_4_) δ 8.25 (s, 2H), 7.67 (s, 1H), 7.65 (s, 2H), 7.16 (s, 1H), 3.82 (t, *J* = 6.8 Hz, 1H), 3.70 (t, *J* = 6.4 Hz, 2H), 2.50 (q, *J* = 6.4 Hz, 2H) ppm. HRMS calculated for C_19_H_13_Cl_3_N_2_O_2_F_6_ (M − H)^−^: 519.9947. Found: 519.9946.

*2-(2-chloroethyl)-N^1^-(3,5-dichlorophenyl)-N^3^-(4-nitro-3-(trifluoromethyl)phenyl)malonamide* (**29**): ^1^H-NMR (400 MHz, MeOD-*d*_4_) δ 8.24 (s, 1H), 8.04 (q, *J* = 8.8 Hz, 2H), 7.63 (s, 2H), 7.14 (s, 1H), 3.85 (t, *J* = 6.8 Hz, 1H), 3.70 (t, *J* = 6.8 Hz, 2H), 2.49 (q, *J* = 6.8 Hz, 2H) ppm. HRMS calculated for C_18_H_13_Cl_3_N_3_O_4_F_3_ (M − H)^−^: 496.9924. Found: 496.9928.

#### 4.2.3. General Procedure for the Synthesis of **30** and **31**

To a mixture of thionyl chloride (14 equiv.) without any solution, cyclopropane-1,1-dicarboxylic acid (1 equiv.) was slowly added, and the reaction mixture was stirred at 80 °C for 16 h. The intermediate was cooled to ambient temperature and concentrated under reduced pressure. A mixture of aniline derivative (2.5 equiv.) and pyridine (1 equiv.) in anhydrous THF was added dropwise to an ice-cold solution of the intermediate in anhydrous THF (15–20 mL). The reaction mixture was stirred at ambient temperature for 2 h. The product was extracted with ethyl acetate three times and the combined organic extracts were washed with brine, dried over MgSO_4_, and concentrated. The crude mixture was purified by flash column chromatography (EtOAc/hexane = 1/3 to 1/2) to give **30** and **31** (yield: 2–31%).

*N,N′-Bis(4-chloro-3-(trifluoromethyl)phenyl)cyclopropane-1,1-dicarboxamide* (**30**): ^1^H-NMR (400 MHz, MeOD-*d*_4_) δ 8.12 (d, *J* = 2.8 Hz, 2H), 7.78 (dd, *J* = 8.4, 2.8 Hz, 2H), 7.51 (d, *J* = 8.4 Hz, 2H), 1.63 (s, 4H) ppm. ^13^C-NMR (100 MHz, MeOD-*d*_4_) δ 171.0, 138.9, 132.9, 129.2 (q, *J* = 31.1 Hz), 127.5, 126.2, 124.1 (q, *J* = 270.7 Hz), 120.8 (q, *J* = 5.6 Hz), 31.8, 17.6 ppm. HRMS calculated for C_19_H_12_Cl_2_F_6_N_2_O_2_ (M − H)^−^: 483.0096. Found: 483.0102.

*N,N′-Bis(3-ethynylphenyl)cyclopropane-1,1-dicarboxamide* (**31**): ^1^H-NMR (400 MHz, MeOD-*d*_4_) δ 7.75 (s, 2H), 7.53 (d, *J* = 8.0 Hz, 2H), 7.30 (t, *J* = 8.0 Hz, 2H), 7.22 (d, *J* = 8.0 Hz, 2H), 3.48 (s, 2H), 1.62 (s, 4H) ppm. HRMS calculated for C_21_H_16_N_2_O_2_ (M − H)^−^: 327.1128. Found: 327.1131.

#### 4.2.4. General Procedure for the Synthesis of **9**

Allyl bromide or benzyl bromide (1 equiv.) was slowly added to a mixture of diethyl malonate (1.5 equiv.) and K_2_CO_3_ (3.0 equiv.) in acetone solution, and the reaction mixture was stirred at room temperature for 24 h. The reaction was concentrated, neutralized with NH_4_Cl, and extracted with CH_2_Cl_2_. The combined organic extracts were washed with brine, dried over MgSO_4_, and concentrated. The crude mixture was purified by flash column chromatography (EtOAc/hexane = 1/10 to 1/9) to give **9** (yield: 24–74%).

#### 4.2.5. General Procedure for the Synthesis of **10**

KOH (2.0 equiv.) was slowly added to a mixture of 9 (1.0 equiv.) in the ethanol solution, and the reaction mixture was stirred at 85 °C for 2 h. The reaction was concentrated, neutralized with 5 M HCl, and extracted with EtOAc. The organic extract was dried over MgSO_4_ and concentrated (yield: 52–81%).

#### 4.2.6. General Procedure for the Synthesis of **32**–**39**

Compound **10** (1 equiv.) was slowly added to a mixture of thionyl chloride (14 equiv.) without any solution, and the reaction mixture was stirred at 80 °C for 16 h. The intermediate was cooled to ambient temperature and concentrated under reduced pressure. A mixture of aniline derivative (2.5 equiv.) and pyridine (1 equiv.) in anhydrous THF was added dropwise to an ice-cold solution of the intermediate in anhydrous THF (15–20 mL). The reaction mixture was stirred at ambient temperature for 2 h. The product was extracted with ethyl acetate three times and the combined organic extracts were washed with brine, dried over MgSO_4_, and concentrated. The crude mixture was purified by flash column chromatography (EtOAc/hexane = 1/5 to 1/1) to give **32**–**39** (yield: 8–20%).

*2-Allyl-N^1^,N^3^-bis(4-chloro-3-(trifluoromethyl)phenyl)malonamide* (**32**): ^1^H-NMR (400 MHz, CDCl_3_-*d*_1_) δ 9.08 (s, 2H), 7.93 (s, 2H), 7.67 (d, *J* = 8.4 Hz, 2H), 7.45 (d, *J* = 8.4 Hz, 2H), 5.81–5.73 (m, 1H), 5.19 (d, *J* = 16.8 Hz, 1H), 5.12 (d, *J* = 9.6 Hz, 1H), 3.39 (t, *J* = 7.6 Hz, 1H), 2.79 (t, *J* = 7.6 Hz, 2H) ppm. ^13^C-NMR (100 MHz, MeOD-*d*_4_) δ 169.9, 138.8, 135.4, 133.0, 129.3 (q, *J* = 31.1 Hz), 127.4, 125.5, 124.1 (q, *J* = 270.7 Hz), 120.0 (q, *J* = 5.4 Hz), 118.3, 56.4, 35.9 ppm. HRMS calculated for C_20_H_14_Cl_2_N_2_O_2_F_6_ (M − H)^−^: 498.0337. Found: 498.0338.

*2-Allyl-N^1^,N^3^-bis(3-ethynylphenyl)malonamide* (**33**): ^1^H-NMR (400 MHz, MeOD-*d*_4_) δ 7.74 (s, 2H), 7.55 (d, *J* = 7.6 Hz, 2H), 7.28 (t, *J* = 7.6 Hz, 2H), 7.20 (d, *J* = 7.6 Hz, 2H), 5.93–5.83 (m, 1H), 5.18 (d, *J* = 17.2 Hz, 1H), 5.08 (d, *J* = 10.4 Hz, 1H), 3.54 (t, *J* = 7.2 Hz, 1H), 3.47 (s, 2H), 2.77 (t, *J* = 7.2 Hz, 2H) ppm. ^13^C-NMR (100 MHz, MeOD-*d*_4_) δ 169.9, 139.5, 135.5, 130.0, 129.0, 124.6, 124.2, 121.8, 118.2, 84.0, 78.8, 56.2, 36.3 ppm. HRMS calculated for C_22_H_18_N_2_O_2_S_2_ (M − H)^−^: 342.1368. Found: 342.1364.

*2-Allyl-N^1^,N^3^-bis(3-ethylphenyl)malonamide* (**34**): ^1^H-NMR (400 MHz, CDCl_3_-*d*_1_) δ 8.83 (s, 2H), 7.37 (d, *J* = 7.6 Hz, 2H), 7.36 (s, 2H), 7.21 (t, *J* = 7.6 Hz, 2H), 6.96 (d, *J* = 7.6 Hz, 2H), 5.87–5.79 (m, 1H), 5.18 (d, *J* = 16.8 Hz, 1H), 5.08 (d, *J* = 10.4 Hz, 1H), 3.39 (t, *J* = 7.2 Hz, 1H), 2.79 (t, *J* = 7.2 Hz, 2H), 2.61 (q, *J* = 7.6 Hz, 4H), 1.20 (t, *J* = 7.6 Hz, 6H) ppm. HRMS calculated for C_22_H_26_N_2_O_2_ (M − H)^−^: 350.1994. Found: 350.2003.

*2-Allyl-N^1^,N^3^-bis(3,5-dichlorophenyl)malonamide* (**35**): ^1^H-NMR (400 MHz, CDCl_3_-*d*_1_) δ 8.86 (s, 2H), 7.50 (d, *J* = 1.6 Hz, 4H), 7.13 (t, *J* = 1.6 Hz, 2H), 5.81–5.73 (m, 1H), 5.20 (d, *J* = 17.2 Hz, 1H), 5.14 (d, *J* = 10.4 Hz, 1H), 3.35 (t, *J* = 7.2 Hz, 1H), 2.77 (t, *J* = 7.2 Hz, 2H) ppm. HRMS calculated for C_18_H_14_Cl_4_N_2_O_2_ (M − H)^−^: 429.9809. Found: 429.9818.

*2-Benzyl-N^1^,N^3^-bis(4-chloro-3-(trifluoromethyl)phenyl)malonamide* (**36**): ^1^H-NMR (400 MHz, MeOD-*d*_4_) δ 7.98 (d, *J* = 2.0 Hz, 2H), 7.79 (dd, *J* = 8.8, 2.0 Hz, 2H), 7.55 (d, *J* = 8.8 Hz, 2H), 7.29–7.25 (m, 5H), 3.71 (s, 2H) ppm. ^13^C-NMR (100 MHz, MeOD-*d*_4_) δ 167.3, 138.2, 135.4, 132.9, 131.9, 129.3 (q, *J* = 31.2 Hz), 129.2, 128.7, 128.3, 126.8, 124.1 (q, *J* = 270.9 Hz), 121.4 (q, *J* = 5.6 Hz), 74.1, 45.1 ppm. HRMS calculated for C_24_H_16_Cl_2_N_2_O_2_F_6_ (M − H)^−^: 548.0493. Found: 548.0500.

*2-Benzyl-N^1^,N^3^-bis(3,5-dichlorophenyl)malonamide* (**37**): ^1^H-NMR (400 MHz, MeOD-*d*_4_) δ 7.57 (s, 4H), 7.28 (s, 2H), 7.25–7.15 (m, 5H), 3.71 (t, *J* = 8.0 Hz, 1H), 3.32 (d, *J* = 8.0 Hz, 2H) ppm. HRMS calculated for C_22_H_16_Cl_4_N_2_O_2_ (M − H)^−^: 479.9966. Found: 479.9960.

*2-Benzyl-N^1^,N^3^-bis(4-nitro-3-(trifluoromethyl)phenyl)malonamide* (**38**): ^1^H-NMR (400 MHz, MeOD-*d*_4_) δ 8.19 (s, 2H), 8.02 (s, 4H), 7.32–7.17 (m, 5H), 3.38 (s, 2H) ppm. ^13^C-NMR (100 MHz, DMSO-*d*_6_) δ 167.8, 143.1, 141.6, 138.2, 128.6, 128.3, 127.6, 126.4, 123.0, 122.7, 121.9 (q, *J* = 271.5 Hz), 117.5 (q, *J* = 5.6 Hz), 56.7, 34.3 ppm. HRMS calculated for C_24_H_16_N_4_O_6_F_6_ (M − H)^−^: 570.0974. Found: 570.0975.

*2-Benzyl-N^1^,N^3^-bis(3,5-bis(trifluoromethyl)phenyl)malonamide* (**39**): ^1^H-NMR (400 MHz, CDCl_3_-*d*_1_) δ 9.30 (s, 2H), 7.91 (s, 4H), 7.66 (s, 2H), 7.17–7.06 (m, 5H), 3.57 (t, *J* = 8.0 Hz, 1H), 3.34 (d, *J* = 8.0 Hz, 2H) ppm. ^13^C-NMR (100 MHz, DMSO-*d*_6_) δ 167.7, 140.5, 138.3, 130.7 (q, *J* = 32.5 Hz), 128.7, 128.3, 126.5, 123.1 (q, *J* = 271.2 Hz), 119.1, 116.5, 56.7, 34.4 ppm. HRMS calculated for C_26_H_16_N_2_O_2_F_12_ (M − H)^−^: 616.1020. Found: 616.1027.

### 4.3. Biological Assay

Biofilm eradication synergy assay. The interaction of **26** and antibiotics commonly used for eradicating MRSA in the biofilm was evaluated with an assay modified from the minimal biofilm eradication concentration (MBEC) assay [15] and the microdilution checkerboard assay. Briefly, MRSA (ATCC 33591) grown overnight was suspended in cation-adjusted MH (CAMH) broth to a concentration of 5 × 10^5^ cfu/mL and aliquoted into the wells of a 96-well plate. Then, the plate was covered by a sterile peg lid and incubated at 37 °C for 24 h. The biofilm formed on the pegs was rinsed with sterile PBS twice to remove loosely adherent planktonic cells followed by exposing to **26** and test antibiotic at two-fold escalating concentrations, ranging from 1 to 64 mg/L, in a new 96-well plate for 24 h. Next, the peg lid was rinsed with PBS twice, transferred to another 96-well plate with 200 μL of CAMH in the wells, and sonicated by using a table sonicator for 5 min. After sonication, the peg lid was replaced by a flat lid, and the 96-well plate was incubated at 37 °C for 24 h. The MBEC was determined as the lowest concentration in which no growth of bacteria from biofilm was observed. The fractional inhibitory concentration for each drug was calculated as follows: FIC_A_ = (MBEC of drug A in the presence of drug B)/(MBEC of drug A alone). Similarly, the FIC of drug B was calculated. The FIC index (FICI) was calculated as: FICI = FIC_A_ + FIC_B_. Synergy was defined by FICI ≤ 0.5, additive by FICI between 0.5 and 1, independently defined by FICI ≥ 1, and antagonism was defined by FICI ≥ 4 [15].

Cell viability assay. The cytotoxicity of individual test agents towards K-562 human erythromyeloblastoid leukemia cell line (BCRC, Hsinchu, Taiwan) was evaluated by using the MTT (Alfa Aesar, Ward Hill, MA, USA) cell viability assay. Briefly, cells were seeded into 96-well plates at 1 × 10^4^ cells/well (with a minimum of six wells per test condition) in cell culture medium supplemented with 10% fetal bovine serum (FBS) (Gibco, Grand Island, NY, USA). After overnight incubation at 37 °C under 5% CO_2_, the medium was removed and replaced with 200 mL of fresh medium containing varying concentrations of compounds dissolved in DMSO (final concentration, 0.1%). After 24 h, the medium was removed again and replaced by 100 mL of 0.5 mg/mL MTT in 10% FBS-containing medium and the cells were incubated in a CO_2_ incubator at 37 °C for 2 h. Subsequently, the medium was removed and the reduced MTT dye in each well was dissolved with 100 mL of DMSO. Absorbance at 570 nm was measured with a microplate reader (VersaMax, Molecular Devices, Sunnyvale, CA, USA). The IC_50_ of each drug was determined from dose–response curves by using CalcuSyn software (Biosoft, Cambridge, UK).

Antibacterial assays. The MIC of each agent was determined following the guidelines of the Clinical and Laboratory Standards Institute (CLSI). For the broth microdilution method, bacteria grown overnight on LB (Athena Enzyme Systems, Baltimore, MD, USA) agar plates were suspended in PBS to an OD of 1.0 at 600 nm (5 × 10^8^ cfu/mL), and then diluted in CAMHB (Difco Laboratories, Detroit, MI, USA) to a final concentration of 5 × 10^5^ cfu/mL. The bacterial suspensions were exposed to the test agents and chloramphenicol at escalating doses, ranging from 0.125 to 64 mg/L, in triplicate in 96-well plates, and the plates were incubated at 37 °C for 24 h. The MIC of each agent was defined as the lowest concentration at which no growth of bacteria was observed.

## Figures and Tables

**Table 1:**
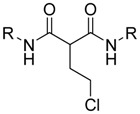
Antibacterial activity of symmetric ethyl chloride malonate derivatives against *S. aureus* NCTC8325.

Cpd	R	*S. aureus* NCTC8325 MIC (mg/L)	Cpd	R	*S. aureus* NCTC8325 MIC (mg/L)
**13**	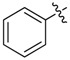	>8	**20**	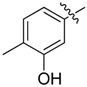	8
**14**	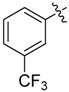	2	**21**	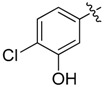	16
**15**	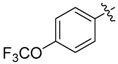	1	**22**	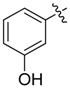	>64
**16**	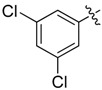	0.25	**23**	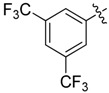	32
**17**	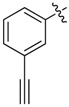	>8	**24**	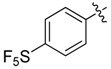	32
**18**	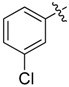	>8	**25**	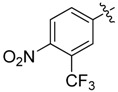	1
**19**	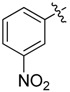	>64	**26**	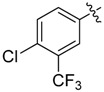	0.5

**Table 2:**
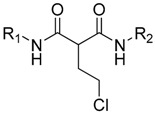
Antibacterial activity of asymmetric ethyl chloride malonate derivatives against *S. aureus* NCTC8325.

Cpd	R_1_	R_2_	*S. aureus* NCTC8325 MIC (mg/L)
**27**	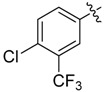	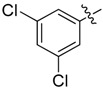	0.25
**28**	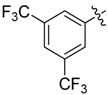	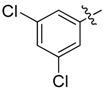	0.25
**29**	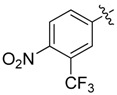	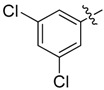	0.5

**Table 3:**
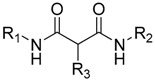
Antibacterial activity of cyclopropanyl-, allyl- and benzyl-substituted malonate derivatives against *S. aureus* NCTC8325.

Cpd	R_1_	R_2_	R_3_	*S. aureus* NCTC8325 MIC (mg/L)
**30**	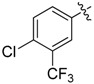	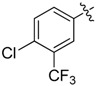	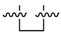	>8
**31**	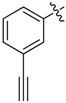	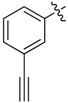	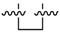	8
**32**	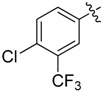	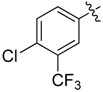	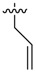	8
**33**	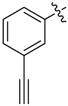	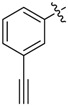	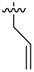	>32
**34**	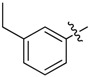	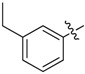	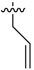	>32
**35**	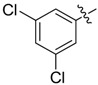	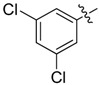	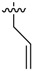	>32
**36**	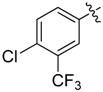	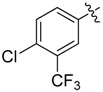	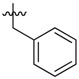	2
**37**	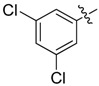	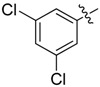	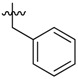	2
**38**	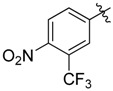	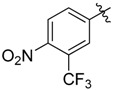	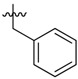	0.5
**39**	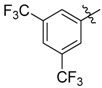	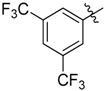	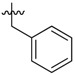	4

**Table 4:** Comparison of antibacterial activity of test agents against *S. aureus* NCTC8325 and MRSA ATCC33592.

Cpd	*S. aureus* NCTC8325 MIC (mg/L)	MRSA ATCC 33592 MIC (mg/L)
**33**	>32	>32
**26**	0.5	0.5
**27**	0.25	0.25
**28**	0.5	0.5
**29**	0.5	0.5
**36**	2	2
**37**	2	2
**38**	0.5	0.5
**39**	4	4

**Table 5:** Antibacterial activity of **26**, oxacillin and vancomycin against *S. aureus*, MRSA, VISA (vancomycin-intermediate *Staphylococcus aureus*), VRSA (vancomycin-resistant *Staphylococcus aureus*) and 50 clinical MRSA isolates; the selectivity ratio of **26** by IC_50_ in K562 cells versus MIC in *S. aureus* NCTC8325. NTUH, National Taiwan University Hospital.

Cpd	MIC (mg/L)				MIC_90_ (mg/L)	Selectivity Ratio in K562 Cells
	*S. aureus* NCTC8325	MRSA ATCC33592	VISA (NTUH Isolate)	VRSA (SJC1200)	50 NTUH MRSA Isolates	
26	0.5	0.5	0.5	0.5	0.5	40.46
Oxacillin	0.125	>256	-	-	-	-
Vancomycin	1	-	4	512	-	-

Selectivity ratio: IC_50_ of K562/ MIC of *S. aureus.*

**Table 6:** Antibacterial activity of **26** in combination with common antibiotics against MRSA in the biofilm. MBEC, minimum biofilm eradication concentration. FICI, fractional inhibitory concentration index.

Combinations		MRSA ATCC 33591			
		MBEC (mg/L)		FICI	Outcome
		Alone	Combined		
1	**26**	16	16	2	Independent
	Vancomycin	>64	>64		
2	**26**	8	8	2	Independent
	Gentamycin	>64	>64		
3	**26**	8	8	2	Independent
	Ampicillin	>64	>64		
4	**26**	16	16	2	Independent
	Ofloxacin	>64	>64		
5	**26**	16	16	2	Independent
	Trimethoprim	>64	>64		
6	**26**	16	16	2	Independent
	Tetracycline	>64	>64		
7	**26**	8	8	2	Independent
	Erythromycin	>64	>64		
8	**26**	32	32	2	Independent
	Daptomycin	>64	>64		
9	**26**	16	16	2	Independent
	Linezolid	>64	>64		
10	**26**	16	16	2	Independent
	Chloramphenicol	>64	>64		
11	**26**	8	1	0.375	Synergy
	Rifampicin	8	2		

## References

[1] Ventola C.L. (2015). The antibiotic resistance crisis: Part 1: Causes and threats. Pharm. Ther..

[2] Kirby T. (2015). New antibiotic development hailed as game changing. Lancet Infect. Dis..

[3] World Health Organization (2017). Antibacterial Agents in Clinical Development.

[4] Chambers H.F. (1997). Methicillin resistance in staphylococci: Molecular and biochemical basis and clinical implications. Clin. Microbiol. Rev..

[5] Liu C., Bayer A., Cosgrove S.E., Daum R.S., Fridkin S.K., Gorwitz R.J., Kaplan S.L., Karchmer A.W., Levine D.P., Murray B.E. (2011). Clinical practice guidelines by the infectious diseases society of america for the treatment of methicillin-resistant *Staphylococcus aureus* infections in adults and children. Clin. Infect. Dis..

[6] Lowy F.D. (1998). Staphylococcus aureus infections. N. Engl. J. Med..

[7] Jerwood S., Cohen J. (2008). Unexpected antimicrobial effect of statins. J. Antimicrob. Chemother..

[8] Liu C.I., Liu G.Y., Song Y., Yin F., Hensler M.E., Jeng W.Y., Nizet V., Wang A.H., Oldfield E. (2008). A cholesterol biosynthesis inhibitor blocks *Staphylococcus aureus* virulence. Science.

[9] Amaral L., Viveiros M., Molnar J. (2004). Antimicrobial activity of phenothiazines. In Vivo.

[10] Abbate E., Vescovo M., Natiello M., Cufre M., Garcia A., Gonzalez Montaner P., Ambroggi M., Ritacco V., van Soolingen D. (2012). Successful alternative treatment of extensively drug-resistant tuberculosis in argentina with a combination of linezolid, moxifloxacin and thioridazine. J. Antimicrob. Chemother..

[11] Su J.C., Mar A.C., Wu S.H., Tai W.T., Chu P.Y., Wu C.Y., Tseng L.M., Lee T.C., Chen K.F., Liu C.Y. (2016). Disrupting VEGF-A paracrine and autocrine loops by targeting SHP-1 suppresses triple negative breast cancer metastasis. Sci. Rep..

[12] Chang H.C., Huang Y.T., Chen C.S., Chen Y.W., Huang Y.T., Su J.C., Teng L.J., Shiau C.W., Chiu H.C. (2016). In vitro and in vivo activity of a novel sorafenib derivative SC5005 against MRSA. J. Antimicrob. Chemother..

[13] Archer N.K., Mazaitis M.J., Costerton J.W., Leid J.G., Powers M.E., Shirtliff M.E. (2011). Staphylococcus aureus biofilms: Properties, regulation, and roles in human disease. Virulence.

[14] Hoiby N., Bjarnsholt T., Givskov M., Molin S., Ciofu O. (2010). Antibiotic resistance of bacterial biofilms. Int. J. Antimicrob. Agents.

[15] Ceri H., Olson M.E., Stremick C., Read R.R., Morck D., Buret A. (1999). The calgary biofilm device: New technology for rapid determination of antibiotic susceptibilities of bacterial biofilms. J. Clin. Microbiol..

